# Network Medicine: A Potential Approach for Virtual Drug Screening

**DOI:** 10.3390/ph17070899

**Published:** 2024-07-06

**Authors:** Mingxuan Ma, Mei Huang, Yinting He, Jiansong Fang, Jiachao Li, Xiaohan Li, Mengchen Liu, Mei Zhou, Guozhen Cui, Qing Fan

**Affiliations:** 1 School of Bioengineering, Zhuhai Campus of Zunyi Medical University, Zhuhai 519000, China; mingxuan_ma33@163.com (M.M.); huangm1217@163.com (M.H.); yintinghe@163.com (Y.H.); lee_ka_chiu@126.com (J.L.); liumengchenlmc@gmail.com (M.L.); zm20240409zm@163.com (M.Z.); 2 Science and Technology Innovation Center, Guangzhou University of Chinese Medicine, Guangzhou 570000, China; fangjs@gzucm.edu.cn; 3 Basic Medical Science Department, Zhuhai Campus of Zunyi Medical University, Zhuhai 519041, China

**Keywords:** network medicine, protein–protein interaction (PPI), network proximity, drug screening, disease gene, drug target, omics

## Abstract

Traditional drug screening methods typically focus on a single protein target and exhibit limited efficiency due to the multifactorial nature of most diseases, which result from disturbances within complex networks of protein–protein interactions rather than single gene abnormalities. Addressing this limitation requires a comprehensive drug screening strategy. Network medicine is rooted in systems biology and provides a comprehensive framework for understanding disease mechanisms, prevention, and therapeutic innovations. This approach not only explores the associations between various diseases but also quantifies the relationships between disease genes and drug targets within interactome networks, thus facilitating the prediction of drug–disease relationships and enabling the screening of therapeutic drugs for specific complex diseases. An increasing body of research supports the efficiency and utility of network-based strategies in drug screening. This review highlights the transformative potential of network medicine in virtual therapeutic screening for complex diseases, offering novel insights and a robust foundation for future drug discovery endeavors.

## 1. Introduction

The functionality of living systems depends on complex interactions between molecular components within and across cells. Disease development is rarely caused by single gene abnormalities but, rather, by disturbances in complex networks within and between cells across tissues and organ systems [1]. Consequently, drug screening strategies that focus on a single gene or target have limited effectiveness in treating complex diseases. As an emerging research field, network medicine provides a novel approach for comprehensively understanding disease mechanisms through integrating systems biology data with computational tools. It systematically reveals molecular complexity and drug–disease interactions by identifying the most direct interactions between disease modules and drug modules, as well as exploring the molecular relationships across different disease phenotypes [2]. Interestingly, in traditional Chinese medicine, which does not adhere to the conventional concept of “disease”, proteins associated with symptoms may exhibit topological properties similar to those of disease-associated genes within the human protein–protein interaction (PPI) network [3].

The drug repurposing strategy aims to use approved drugs to overcome the shortcomings of high costs and lengthy time consumption associated with traditional drug development [4]. This strategy is pivotal for swiftly and economically identifying potential drugs for various diseases, particularly rare, complex, and emerging infectious diseases [5,6,7]. Using network medicine in drug discovery is crucial for enhancing the efficiency of drug research and development. Studies involving network medicine have revealed the molecular interaction network between diseases and drugs, enabling both the identification of new indications for approved drugs and the discovery of new therapeutic agents for specific diseases, thus accelerating drug discovery and development [8,9,10]. The growing number of studies employing network medicine methods for new drug discovery showcases the vast potential of network medicine applications.

This review aims to summarize the recent progress in network medicine, focusing on its principles, data preparation methods, and applications for virtual drug screening. This study underscores the crucial role of network medicine in virtual drug and active ingredient screening for various diseases and provides new insights for future drug research and development.

## 2. Principle of Network Medicine-Based Virtual Drug Screening

### 2.1. Network-Based Virtual Single Drug Screening

**Figure 1:**
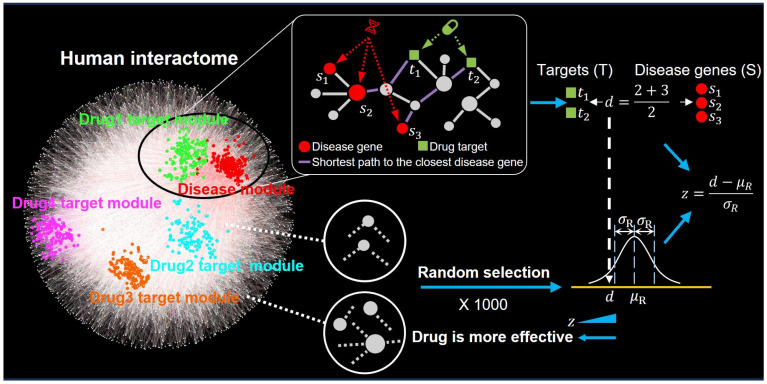
Schematic diagram illustrating the principle of drug screening using network proximity calculation.

In network medicine, complex cellular systems are simplified to consider their components, such as proteins, RNA molecules, and gene sequences, as nodes, while the physical, biochemical, and functional interactions between these components are considered as edges. This approach forms interactome networks, which are networks of interactions between cellular components [11]. In network medicine-based virtual drug screening, “network proximity” is a key concept for quantifying the interactions between drug–target datasets and disease–gene datasets within the interactome [12]. With this concept, the efficacy of a drug is evaluated based on the proximity of its targets (depicted as green squares) to the disease module (depicted as a group of red circles), as shown in Figure 1, which provides a schematic overview of the principle underlying drug screening using the network proximity method. Specifically, closer proximity indicates a potential increase in drug efficacy against the disease. For instance, “Drug Target Module 1” is closer to the “Disease Gene Module” than other drug modules, suggesting greater potential efficacy. To accurately assess the drug–disease relationship while minimizing the impact of node quantity and path diversity within the network, a relative distance measurement method is employed for quantitative analysis. This method involves establishing a reference distance distribution through randomly selecting two paired groups of nodes within the human PPI network while considering the quantity and interconnections of the nodes in each group. This approach has yielded statistically significant differences in drug–disease distances, providing a robust framework for predicting potential therapeutic agents.

The reference distribution is generated by repeating the selection of two paired groups of nodes (e.g., 1000 times). Subsequently, the mean and standard deviation of this distribution were calculated, and the observed drug–disease distance was converted into a standardized *Z*-score. A positive *Z*-score indicates that the observed distance is above the average, whereas a negative *Z*-score means it is below the average. A lower *Z*-score suggests a closer proximity between the drug and the disease. When analyzing drug screening results, drugs are usually ranked in ascending order by their *Z*-scores. This prioritization favors drugs with lower *Z*-scores, suggesting that they have greater potential for experimental validation [12,13].

Several methodologies are available to determine the drug–disease distance within interactome networks. Albert-László Barabási et al. proposed an unsupervised, unbiased network-based framework for assessing the associations between drug targets and diseases. Their study compared five quantified methods for assessing drug–disease relationships by measuring their distance in the human interactome network [12]. Specifically, the average shortest distance (*d_s_*) represents the average distance from each drug target to all the genes’ networks associated with the disease; the closest distance (*d_c_*) measures the average distance from each drug target to the nearest disease gene; the kernel distance (*d_k_*) incorporates the distance from each drug target to all disease genes while exponentially reducing the weight of longer distances; the center distance (*d_cc_*) is defined as the average distance from each drug target to the node of the disease module with the maximum closeness centrality; and the separation distance (*d_ss_*) is calculated by first adding the average distance from each drug target to the nearest disease gene and from each disease gene to the nearest drug target. Then, half of the sum of the average shortest distances between drug targets and between disease genes was subtracted. Among these methods, *d_c_* demonstrated superior accuracy in identifying known drug–disease associations with minimal false positives and was the most accurate in predicting drug–disease correlations. This superiority makes the *d_c_* method the preferred choice for calculating drug–disease distances [12].

### 2.2. Network Medicine Approach for Screening Drug Combinations

**Figure 2:**
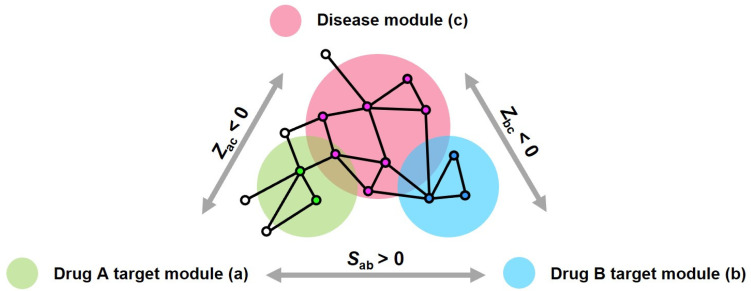
Network proximity principle for two-drug combinations. Two distinct drug target modules, labeled module a and module b, are visually represented by green and blue circles, respectively. A pink circle represents the disease module, with the black lines indicating the interconnections within each module. a and b represent the target modules for drug A and drug B, respectively, while c represents the disease module. The “complementary exposure” pattern between the disease module and each drug target module (*Z_ac_* < 0, *Z_bc_* < 0, *p* < 0.05, and *S_ab_* > 0) suggests that combination therapies would be beneficial for drug development.

Network medicine can also be used to predict drug combinations involving drug pairs that target different components of a disease module (a subgraph of the interactome network). Figure 2 illustrates the interaction between the target modules of two drugs (a and b) and a disease module (c), with the interconnected nodes representing biological entities such as genes or proteins. The “complementary exposure” pattern at *Z_ac_* < 0, *Z_bc_* < 0, *p* < 0.05, and *S_ab_* > 0 indicates a potentially beneficial interaction or synergy between the two drugs when used together instead of separately [14]. This approach applies network analysis to clarify relationships and identify optimal drug combinations.

## 3. The Methodology of Virtual Drug Screening Based on Network Medicine

### 3.1. The Steps of Network Medicine-Based Drug Screening

**Figure 3:**
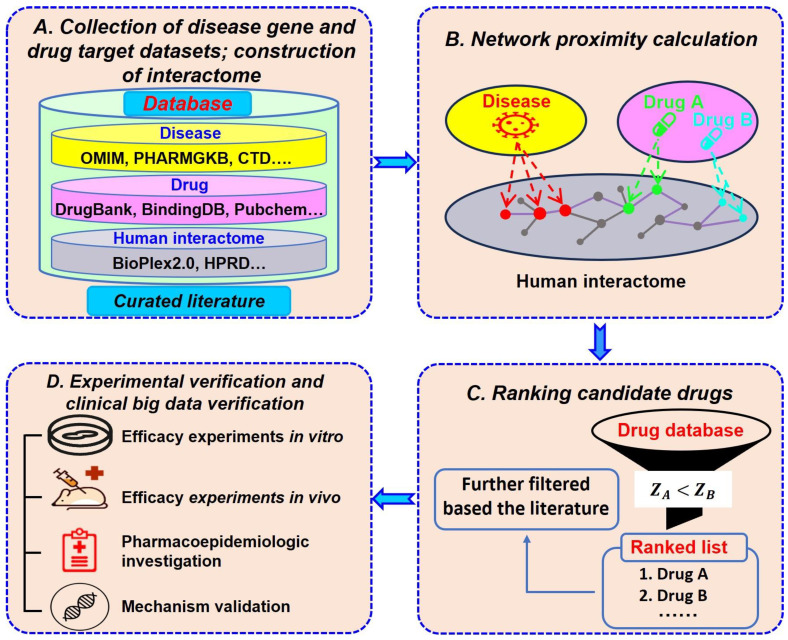
Flow chart of network medicine-based drug screening. (**A**) Drug target, disease gene, and PPI datasets collected from various databases and the literature. (**B**) Network proximity calculations used to analyze the correlations between drugs and diseases. (**C**) Drugs ranked according to the individual *Z*-score. (**D**) Experiments performed to validate the efficacy of the predicted drugs.

The network medicine-based virtual drug screening process comprises four key steps, as illustrated in Figure 3. Initially, datasets incorporating disease-associated genes, drug targets, and the human interactome were compiled from various databases and the scientific literature. This information is collected by one individual and manually curated for completeness and accuracy by two others. The next step involves evaluating the network proximity between the drug target and disease-associated gene modules to predict drug–disease relationships and identify potential effective drugs. Subsequently, the relevant literature and drug data are reviewed to assess the value and feasibility of the candidates. This includes a comprehensive analysis of various factors, such as the pharmacological characteristics, safety, cost-effectiveness, and market potential of the drugs. Only those candidates meeting the criteria were selected for further experimental validation. The fourth step involves a comprehensive validation process, verifying the effectiveness and safety of the candidate drug through multi-level experiments. Typically, this approach includes in vitro (cell-based) experiments, in vivo (animal-based) studies, clinical data validation, and molecular-level experimental investigations, all of which serve to confirm the predicted effects of the candidate drugs.

### 3.2. Construction of the Human Protein–Protein Interaction Network

The distance between drugs and diseases is calculated within the framework of the human interactome network, which is a core tool in network medicine research that enhances our understanding of the molecular mechanisms underlying disease progression [15]. Within this interactome network, proteins serve as nodes that are interconnected by edges, symbolizing their interactions. Due to their pivotal role in regulating metabolic pathways and biological processes, proteins are considered key targets in the development of disease.

Through establishing connections between specific diseases, the protein interactome, and disease-associated subgraphs—termed “disease modules”—researchers have identified distinct biological functions or characteristics [16]. These findings have the potential to enhance our understanding of the molecular mechanisms that contribute to diverse phenotypes and provide new avenues for pharmaceutical interventions in disease treatment. Upon entering the human body, drug molecules interact with specific targets to exert their effects. The relationships between disease genes and drug targets can be elucidated by analyzing the interactome network. The human interactome network is a valuable tool for identifying new disease genes, studying network properties, exploring disease-related subgraphs, and implementing network-based disease classifications [17].

### 3.3. Collection of Disease Gene Datasets

In the process of screening drugs using a network medicine strategy, the accurate and comprehensive identification of disease genes is crucial. Typically, this process begins with database searches, followed by extensive literature reviews to ensure accuracy and comprehensiveness. The main databases (Table 1) for collecting disease genes included the following: ClinVar [18], the Comparative Toxicogenomics Database (CTD) [19], DisGeNET [20], GeneCards [21], genome-wide association studies (GWAS Catalog) [22], the Human Gene Mutation Database (HGMD) [23], the Human Genome Epidemiology (HuGE) Navigator [24], Online Mendelian Inheritance in Man (OMIM) [25], Orphanet [26], the Pharmacogenetics Knowledge Base (PharmGKB) [27], the Therapeutic Target Database (TTD) [28], and the Universal Protein Knowledgebase (UniProt) [29].

Identifying genes related to specific diseases is crucial for researchers to comprehend the potential mechanisms underlying human diseases. This study area poses a significant challenge in biomedical research, prompting researchers to dedicate substantial resources to identifying genes associated with diseases to address this challenge. Nevertheless, an increasing number of scholarly studies indicate that the onset of the majority of human diseases cannot be attributed to a single gene mutation but rather to an intricate interplay between numerous genetic variations and environmental hazards [1]. Researchers have developed multiple databases to study the complex relationships between genes and diseases, with a specific focus on the correlation between phenotypes and genotypes. One such database, OMIM, mainly stores information on genetic diseases and gene mutations [25]. The PharmGKB database compiles data on the impact of human genetic variability in drug response, encompassing details on gene–drug associations, FDA-approved drug labels featuring genetic content, and data from research and clinical trials. Its primary objective is to elucidate the influence of genetic variability on both the efficacy and adverse reactions of drugs [27]. The CTD database serves as a comprehensive resource for investigating the interactions between chemical substances, genes, and diseases. By aggregating data from various studies, insights into the impact of chemical substances on human health have been elucidated [19]. To enhance the comprehensiveness and accuracy of the acquired information, researchers typically employ a combination of multiple databases to corroborate and validate their data [30]. Furthermore, ensuring the reliability of the acquired data requires careful consideration of various factors, including the data source, the intended application, and the accuracy of the data. 

### 3.4. Optimization of the Disease Module

**Figure 4:**
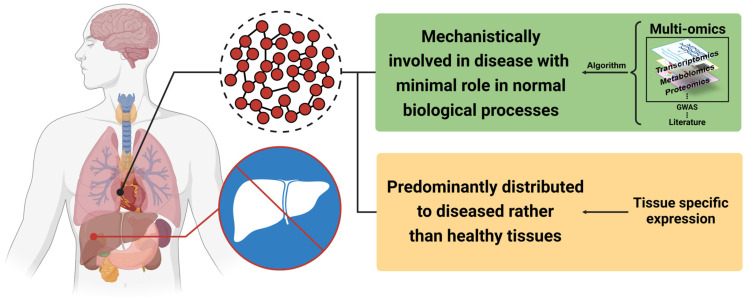
Ideas for optimizing a disease module. This figure was created using BioRender.com and adapted from BioRender.com templates.

In the process of virtual drug screening using network medicine, the quality of the disease module plays a critical role in improving the precision of drug prediction [31]. An ideal disease module should exhibit two fundamental characteristics, as illustrated in Figure 4. The validation of the disease module is crucial and can be conducted using the largest connected component (LCC) method. The LCC *Z*-score is calculated as the difference between the observed LCC size and the mean of randomization. A *Z*-score greater than 1.6 for the LCC indicates that the observed LCC is significantly greater than would be expected by chance [3]. Ideally, the disease module should have significant network proximity to most major pathological endophenotypic networks or biological pathway networks involved in the disease, which demonstrates the accuracy of the disease module construction. In addition, genes within the disease module should be specifically expressed in pathological tissue. Furthermore, the disease module should exhibit a distinct mechanism-regulatory role in the progression of a particular disease, while minimizing its involvement in normal biological processes [32]. Precisely identifying disease-specific genes through omics technologies can optimize the disease module. Additionally, a disease module should be primarily situated in diseased tissue rather than in healthy tissue to reflect specific gene regulation in a pathological state [33]. Within the network medicine framework, optimizing a disease module can facilitate accurate drug prediction and screening by leveraging known disease-specific gene associations or mining omics data.

### 3.5. Collection of Drug and Natural Product Targets

In the process of drug screening under a network medicine framework, in addition to identifying disease-associated genes, identifying the targets of drugs and natural products is critical. Existing drug databases offer a plethora of information encompassing crucial aspects, such as drug molecular structures, biological activity data, protein targets, mechanisms of action, and toxicity profiles. Considering the many small molecules that need to be screened, manually selecting targets from the literature is a laborious task, and it is challenging to collect accurate target information for these molecules. In comparison, sourcing target data from a database proves to be a more efficient approach. Using a drug target database is the preferred method for identifying small molecule drug targets, complemented with manual review and screening, which can enhance the accuracy and reliability of drug target information. Table 2 details various databases that focus on drug and natural product targets, including the Binding Database (BindingDB) [34], the CTD [19], ChEMBL [35], DrugBank [36], DrugCentral [37], Herbal Ingredients Targets (HIT) 2.0 [38], Ingenuity Pathway Analysis (IPA) [39], Integrated Traditional Chinese Medicine (ITCM) [40], The International Union of Basic and Clinical Pharmacology/British Pharmacological Society (IUPHAR/BPS Guide to PHARMACOLOGY) [41], the Protein Data Bank (PDB) [42], PharmGKB [27], PubChem [43], Search Tool for Interactions of Chemicals (STITCH) [44], and TTD [28]. One of the popular databases is DrugBank, which is an open-access resource providing comprehensive information on drugs, their targets, and indications. Given its extensive compilation of FDA-approved drugs, DrugBank is an invaluable resource for research purposes [45].

### 3.6. Calculation of Network Proximity

Calculating network proximity, *d_c_*, involves determining the shortest distance between a drug target and disease genes. This process includes selecting the minimum distance from each drug target to all disease genes and subsequently averaging the minimum distances of all the targets of the drug to derive the *d_c_* value (Equation (1)). Additionally, this can also involve calculating the minimum distances from each disease gene to its nearest drug target, as demonstrated in Equation (2).

(1) $$d_{c}\left(S,T\right)=\frac{1}{∥T∥}\underset{t\in T}{\sum }\underset{s\in S}{\min}d\left(s,t\right)$$

(2) $$d_{c}\left(C,T\right)=\frac{1}{∥C∥+∥T∥}\underset{c\in C}{\sum }\underset{t\in T}{\min}d\left(c,t\right)+\underset{t\in T}{\sum }\underset{c\in C}{\min}d\left(c,t\right)$$

When the *Z*-score is less than 0 and the *p*-value is less than 0.05, the drug identified by virtual screening is highly likely to be effective. To facilitate this calculation within the network medicine framework, we developed an online platform. The calculation platform can be found on the website http://www.zmupredict.cn (accessed on 1 May 2024) for public use [46,47,48]. Users can freely access the platform and submit specified datasets, which are mainly used to provide information on drug targets and disease genes, for network proximity calculations. The platform mainly includes three modules: proximity calculation, subgraph extraction, and combination calculation.

## 4. Application Examples of Network Medicine in Virtual Drug Screening

Traditional drug screening methods are characterized as both time-consuming and costly, with a low likelihood of yielding positive outcomes. In contrast, network medicine offers novel and complementary approaches to drug screening. The use of network medicine in drug screening has increased in recent years, providing fresh perspectives and vigor for drug development research. In this study, we present a compilation of typical virtual drug screening application cases within the network medicine framework. These cases illustrate the efficacy of network medicine in expediting drug development and predicting treatments for complex diseases.

Table 3 showcases various illustrative cases of network medicine-based virtual drug screening. In response to the significant public health threat posed by the coronavirus disease 2019 (COVID-19) epidemic, Albert-László Barabási et al. proposed the use of network medicine to repurpose drugs for COVID-19 treatment. This approach not only fostered hope for a response to the epidemic at that time but also provided important strategies and insights for similar health crises that may break out in the future [49]. The subsequent validation of the drug–response relationship for COVID-19 with patient data revealed that melatonin usage reduced the likelihood of positive SARS-CoV-2 test results by 50–60% [50]. Similarly, using network systems biology to investigate human–virus interactions, researchers found that carvedilol use was significantly associated with a reduced risk of contracting COVID-19 [51]. The potential of network medicine extends beyond infectious diseases. For instance, sildenafil (initially used for hypertension), gemfibrozil (which regulates blood lipids), and pioglitazone (an antidiabetic agent) have been identified as potential treatments for Alzheimer’s disease through pharmacoepidemiologic validation and mechanistic studies in human microglia cells and induced pluripotent stem cells (iPSCs) [52,53,54]. Furthermore, metformin has shown promise in reducing atrial fibrillation in a transcriptomics-based network medicine approach, as validated in human iPSCs-derived atrial-like cardiomyocytes and through pharmacoepidemiologic analysis [55].

In oncology, network proximity analysis identified ouabain as a top candidate for lung adenocarcinoma, demonstrating antitumor effects in non-small cell lung cancer cell lines by targeting the HIF1α/LEO1-mediated metabolic pathway [56]. This finding highlights the utility of network medicine in rapidly identifying new indications for existing drugs. In addition to screening chemical drugs, network medicine has also proven effective in traditional Chinese medicine research. For instance, network medicine has been used to investigate the pharmacodynamic basis and potential molecular mechanisms of various Ziziphi Spinosae Semen formulas used to improve sleep quality [46,57]. These mechanisms involve regulating the Akt/GSK-3β pathway, as revealed by network medicine analysis and experimental validation [57]. It is logical to state that network-based analysis includes examining the signal transduction pathways associated with specific pathologies. Moreover, the functions and underlying mechanisms of foods can be explored using this approach. For instance, a network medicine framework revealed the beneficial effects of tea polyphenols and how rosmarinic acid might prevent cardiovascular diseases through inhibiting platelet aggregation [58]. These discoveries will aid in developing healthy foods and offer a scientific foundation for personalized nutrition and disease prevention. The cases cited herein thoroughly confirm network medicine’s immense potential and broad prospects in drug screening.

**Table 3:** Case examples of virtual drug screening based on network medicine.

Disease/ Symptom Name	Key Finding Information	Validation Methods for the Predicted Results	Ref.
Alzheimer’s disease	Identified sildenafil as a potential treatment using network medicine and data mining methods.	Pharmacoepidemiologic validation, mechanistic observations in human microglia cells and iPSCs.	[53]
	Network-based prediction and retrospective case–control studies revealed gefitinib as a promising candidate for prevention and treatment.	Pharmacoepidemiologic validation.	[54]
Atrial fibrillation	A transcriptomics-based network medicine approach identified metformin as a potential drug candidate.	Validation in human iPSC-derived atrial-like cardiomyocytes and pharmacoepidemiologic analysis.	[55]
Breast cancer and liver cirrhosis	Multiscale interaction network method revealed the therapeutic effects and mechanisms of natural products.	Active ingredient validation in the hepatic stellate cell model.	[59]
Fatty liver	Providing theoretical guidance for studying the pharmacodynamic material basis and action mechanisms of herbal medicine.	Literature-based validation.	[47,48]
Insomnia	Network medicine approach elucidated the active ingredients and molecular mechanisms through which herbal medicine improves sleep.	Literature-based validation.	[46,57]
Non-small cell lung cancer	Genome-wide mapping system using network algorithms facilitated drug repurposing by identifying disease modules from DNA/RNA profiles.	Pharmacoepidemiologic validation, mechanistic assays in non-small cell lung cancer cells.	[56]
Novel coronavirus	Network-based method identified repurposing drug candidates and promising drug combinations against SARS-CoV-2.	Literature-based validation.	[13]
	An integrated method using artificial intelligence, network diffusion, and network proximity repurposes drugs against SARS-CoV-2.	Validation in VeroE6 cells challenged with SARS-CoV-2 virus.	[49]
	An approach using network medicine, clinical insights, and multi-omics analysis highlights melatonin as a potential candidate for preventing and treating COVID-19.	Patient data validation.	[50]
	Provided insights for advancing COVID-19 treatment research through network systems biology.	Validation with patient data and in A549-ACE2 cells challenged with SARS-CoV-2.	[51]
Vascular diseases	Network medicine framework predicted new therapeutic effects of polyphenols.	Validation in platelet.	[58]
Various symptoms	Network proximity between herb targets and symptom modules predicted the herb’s effectiveness in treating symptoms.	Validation with empirical data and patient data.	[3]

These examples not only can assist researchers in swiftly identifying potential therapeutic drug candidates but also offer new perspectives on the mechanisms of drug action.

## 5. Discussion

Network medicine represents a pivotal point in drug screening, with advancements in computational methods and integrative analysis paving new paths in drug discovery. Integrating diverse data types and unraveling complex biological networks offer opportunities to develop innovative preemptive strategies. These efforts are significant, and hold promise for transforming the landscape of disease treatment and drug development. Through incorporating network approaches to predict the therapeutic effects of drugs on diseases, network medicine may provide new insights and directions for personalizing drug repurposing [6,7,60].

Several reviews have explored the realm of network science and its application in drug development, offering valuable insights into this interdisciplinary field. For instance, Cheng et al. focused on biological entities, highlighting the importance of utilizing network paradigms to understand biological interactions and enhance our knowledge of biological systems [61]. Similarly, Paola Paci emphasized the vital role of network analysis in identifying new disease genes, elucidating the biological significance of mutations linked to diseases, and advancing our understanding of these complex systems [62]. Furthermore, a recent study offered a comprehensive overview of the principles of network medicine and its applications in complex kidney disease phenotypes [63]. Nurcan Tuncbag et al. expanded this discourse through exploring network medicine, including conceptual frameworks, practical implementations, and prospective developments [64]. Unlike traditional machine learning methods that rely on drug activity data, network medicine constructs computational models based on the human interactome framework. The predictive accuracy of these models hinges on the precision of drug–target and disease–gene datasets. Notably, recent studies—including an article in the Journal of Chemical Information and Modeling—have emphasized the importance of selecting drugs and their related activity types (e.g., IC_50_ and Ki) for optimal model performance. It was noted that including molecules with poor-quality data can negatively impact a model’s quality [65]. Overall, our review adopts a distinct perspective, focusing on the fundamental principles, methodologies, and exemplary cases related to drug predictability and ranking. This approach highlights the potential of network medicine to revolutionize drug development by leveraging comprehensive and precise biological interaction data.

Based on the principle of network medicine-based drug screening, the validation of predicted outcomes should be conducted through extensive in vivo experiments, including both animal models and human clinical trials. Complex diseases, characterized by systemic biological interactions rather than isolated in vitro mechanisms, require validation within living organisms to accurately ascertain the efficacy and safety of potential therapeutics. The integration of multi-omics data and advanced computational methods provides a comprehensive understanding of the biological networks underlying disease states, thereby improving the identification of viable therapeutic agents [61]. Notably, other analytical techniques such as gene set enrichment analysis and deep learning have shown remarkable efficacy in the analysis of complex biological datasets [13,54]. These methodologies are essential for uncovering hidden patterns and relationships within the data, offering profound insights into disease mechanisms, potential therapeutic targets, and promising drug candidates. Moreover, the application of these methodologies facilitates the translation of high-throughput data into actionable biological insights, thereby bridging the gap between computational predictions and clinical application.

While the applications of network medicine in drug screening are expanding, several challenges need to be addressed. First, the predicted associations between diseases and drugs currently do not distinguish between therapeutic effects and toxic side effects. Second, the precision of drug screening largely depends on both the quality and quantity of disease gene data and available drug targets. A prevalent issue is the incompleteness of databases containing disease genes and drug targets. Therefore, systematically refining these databases with new experimental data is imperative. Third, the contribution weight of an individual target to network medicine-based drug screening remains a subject of ongoing debate. Fourth, in the era of multi-omics, effectively integrating network medicine with various data sources continues to pose challenges that require further investigation [66]. Notably, the incompleteness of the human interactome, particularly concerning the interactomes specific to certain diseases or tissues, poses substantial limitations to the effectiveness of this approach. Fifth, only a few studies account for the fact that interactions and functional modules vary with time and location [67,68]. Future studies should incorporate data on the temporal and spatial dynamics of interactions and functional modules into networks, considering the dynamic nature of interactome networks. Finally, the current methodology for screening epigenetically sensitive drugs through network-based methods is considered inappropriate due to the associated methodological constraints [69]. Although the accuracy of drug screening using network medicine methods needs improvement, its application and identification of drugs that show promising efficacy in both in vitro and in vivo experiments are increasing, indicating its vast potential in virtual drug screening, particularly for complex diseases and emerging infectious diseases.

## Figures and Tables

**Table 1:** Databases for collecting disease genes.

Database Name	Relevant Key Information	URL	Ref.
ClinVar	Focuses on gene–disease associations and gene expression and interactions	https://www.ncbi.nlm.nih.gov/clinvar	[18]
CTD	Contains relationships between diseases and genes	http://ctdbase.org	[19]
DisGeNET	Collects genes and variants associated with human diseases	https://www.disgenet.org	[20]
GeneCards	Provides detailed gene–disease associations and gene-related information	https://www.genecards.org	[21]
GWAS Catalog	Provides SNP–trait associations from published genome-wide association studies	https://www.ebi.ac.uk/gwas	[22]
HGMD	Collects gene lesions responsible for human inherited diseases	https://www.hgmd.cf.ac.uk/ac/index.php	[23]
HuGE Navigator	Covers prevalence of genetic variation, gene–disease associations, and gene interactions	https://phgkb.cdc.gov/PHGKB/hNHome.action	[24]
OMIM	Contains information on the relationships between human genes and diseases	https://www.omim.org	[25]
Orphanet	Focuses on rare diseases and associated genetic data	https://www.orpha.net/consor/cgi-bin/index.php	[26]
PharmGKB	Describes the associations between diseases and genetic data in detail	https://www.pharmgkb.org	[27]
TTD	Provides comprehensive data on therapeutic targets and their associations with diseases	https://db.idrblab.net/ttd	[28]
UniProt	Provides comprehensive information about protein function and aids in understanding the genes associated with specific diseases	https://www.uniprot.org	[29]

All URLs in the table were accessed on 2 July 2024.

**Table 2:** Databases for collecting the targets of drugs and natural products.

Database Name	Relevant Key Information	URL	Ref.
BindingDB	Database of experimentally determined protein–ligand binding affinities	https://www.bindingdb.org/bind/index.jsp	[34]
CTD	Chemical–gene/protein interactions	https://ctdbase.org	[19]
ChEMBL	Drug target information	https://www.ebi.ac.uk/chembl	[35]
DrugBank	Drug target information	https://go.drugbank.com	[36]
DrugCentral	Drugs and target mechanisms of action	https://drugcentral.org	[37]
HIT 2.0	Information on herbal ingredient–target interactions	http://www.badd-cao.net:2345/	[38]
IPA	Binding protein information	https://www.ingenuity.com	[39]
ITCM	Manually organized component targets	http://itcm.biotcm.net/	[40]
IUPHAR/BPS Guide to PHARMACOLOGY	Comprehensive data on drug targets	https://www.guidetopharmacology.org	[41]
PDBbind	Protein–ligand binding affinity 3D structural data	http://pdbbind.org.cn/	[42]
PharmGKB	Genetic variations that affect drug response	https://www.pharmgkb.org	[27]
PubChem	Genes/proteins that interact with the compound	https://pubchem.ncbi.nlm.nih.gov	[43]
STITCH	Chemical and protein interactions	http://stitch.embl.de/	[44]
TTD	Drugs and therapeutic targets	https://db.idrblab.net/ttd	[28]

All URLs in the table were accessed on 2 July 2024.

## Data Availability

The relevant codes used during the current study are available from the corresponding authors upon reasonable request.
